# A quantitative analysis of the acidosis of cardiac arrest: a prospective observational study

**DOI:** 10.1186/cc3714

**Published:** 2005-05-23

**Authors:** Jun Makino, Shigehiko Uchino, Hiroshi Morimatsu, Rinaldo Bellomo

**Affiliations:** 1 Staff specialist in emergency, Tertiary Emergency Medical Center, Tokyo Metropolitan Bokuto Hospital, Tokyo, Japan; 2 Staff specialist in intensive care, Department of Emergency and Critical Care Medicine, Saitama Medical Center, Saitama Medical School, Saitama, Japan; 3 Staff specialist in intensive care, Department of Anesthesiology and Resuscitology, Okayama University Medical School, Okayama, Japan; 4 Director of intensive care research, Department of Intensive Care and Department of Medicine, Austin & Repatriation Medical Centre, Melbourne, Australia

## Abstract

**Introduction:**

Metabolic acidosis is common in patients with cardiac arrest and is conventionally considered to be essentially due to hyperlactatemia. However, hyperlactatemia alone fails to explain the cause of metabolic acidosis. Recently, the Stewart–Figge methodology has been found to be useful in explaining and quantifying acid–base changes in various clinical situations. This novel quantitative methodology might also provide useful insight into the factors responsible for the acidosis of cardiac arrest. We proposed that hyperlactatemia is not the sole cause of cardiac arrest acidosis and that other factors participate significantly in its development.

**Methods:**

One hundred and five patients with out-of-hospital cardiac arrest and 28 patients with minor injuries (comparison group) who were admitted to the Emergency Department of a tertiary hospital in Tokyo were prospectively included in this study. Serum sodium, potassium, ionized calcium, magnesium, chloride, lactate, albumin, phosphate and blood gases were measured as soon as feasible upon arrival to the emergency department and were later analyzed using the Stewart–Figge methodology.

**Results:**

Patients with cardiac arrest had a severe metabolic acidosis (standard base excess -19.1 versus -1.5; *P *< 0.0001) compared with the control patients. They were also hyperkalemic, hypochloremic, hyperlactatemic and hyperphosphatemic. Anion gap and strong ion gap were also higher in cardiac arrest patients. With the comparison group as a reference, lactate was found to be the strongest determinant of acidosis (-11.8 meq/l), followed by strong ion gap (-7.3 meq/l) and phosphate (-2.9 meq/l). This metabolic acidosis was attenuated by the alkalinizing effect of hypochloremia (+4.6 meq/l), hyperkalemia (+3.6 meq/l) and hypoalbuminemia (+3.5 meq/l).

**Conclusion:**

The cause of metabolic acidosis in patients with out-of-hospital cardiac arrest is complex and is not due to hyperlactatemia alone. Furthermore, compensating changes occur spontaneously, attenuating its severity.

## Introduction

Metabolic acidosis is common in patients with cardiac arrest and is conventionally considered to be due essentially to hyperlactatemia [1-6]. However, hyperlactatemia alone fails to explain the cause of metabolic acidosis in some patients [3]. Traditional measures using the anion gap, standard bicarbonate and standard base excess might help to understand this acidosis [7,8]. However, they give little information about the mechanisms involved and the quantitative contribution of each variable [9-12], especially in the presence of major changes in serum electrolytes and albumin concentration.

Recently, the Stewart–Figge methodology [13,14] has been found to be useful in explaining and quantifying acid–base changes in clinical situations in which conventional analysis was deficient [15-18]. This novel quantitative methodology might also provide useful insight into the factors responsible for the acidosis of cardiac arrest.

We proposed that hyperlactatemia is not the sole cause of cardiac arrest acidosis and that other factors participate significantly in its development. We tested this hypothesis by conducting a prospective study of patients admitted to the Emergency Department of a tertiary hospital in Tokyo, Japan, and by applying quantitative principles to the assessment of their acid–base disorders.

## Materials and methods

This study took place in an Emergency Department of a tertiary hospital in Tokyo, Japan. We prospectively examined out-of-hospital patients with cardiac arrest admitted to the department from May 2003 to October 2003. Because of the anonymous and non-interventional manner of the study, informed consent was not obtained.

Cardiac arrest was defined as the absence of both spontaneous respiration and palpable pulse. Cardiac arrest was described as witnessed arrest if the collapse of a patient was witnessed by a bystander or the emergency ambulance service. All patients were resuscitated in accordance with the Guidelines 2000 for Cardiopulmonary Resuscitation and Emergency Cardiovascular Care [19]. Data evaluated included age, sex, initial electrocardiographic rhythm at the scene, and possible cause. To compare the acid–base characteristics of these patients, we used a comparison group consisting of patients with minor injuries who were discharged within 2 days after admission. We used this group of patients as a comparison group because we routinely measured all variables required for the analysis such as lactate and phosphate in these patients.

Arterial samples were collected in heparinized plastic syringes and analyzed with a blood-gas analyzer (ABL 725; Radiometer, Copenhagen, Denmark) at the time of admission. The analyzer measured samples at 37°C. We collected the following data from the analyzer output: pH, partial pressure of carbon dioxide, bicarbonate, standard base excess, lactate and ionized calcium. Blood samples were also analyzed at the hospital central laboratory for the measurement of multiple biochemical variables including sodium, potassium, total magnesium, chloride, albumin and phosphate (Hitachi 7350 and 7360; Hitachi Industry, Tokyo, Japan). No sodium bicarbonate was administered before blood sampling.

### Conceptual framework for the interpretation of quantitative acid–base analysis

Quantitative physicochemical analysis of the results was performed with Stewart's quantitative biophysical methods [13] as modified by Figge and colleagues [14] to take into account the effects of plasma proteins. This method involves first calculating the apparent strong ion difference (SID_a_):

SID_a _= [Na^+^] + [K^+^] + [Mg^2+^] + [Ca^2+^] - [Cl^- ^] - [lactate^- ^]

(all concentrations in meq/l).

However, this equation does not take into account the role of weak acids (CO_2_, albumin and phosphate) in the balance of electrical charges in plasma water. This is expressed through the calculation of the effective strong ion difference (SID_e_). The formula for SID_e _as determined by Figge and colleagues [14] is as follows:

SID_e _= 1000 × 2.46 × 10^-11^× *P*_CO2_/(10^-pH^) + [albumin] × (0.123 × pH - 0.631) + [phosphate] × (0.309 × pH - 0.469)

In this equation, *P*_CO2 _(the partial pressure of CO_2_) is measured in mmHg, albumin in g/l, and phosphate in mmol/l. This formula accounts quantitatively for the contribution of weak acids to the electrical charge equilibrium in plasma.

Once weak acids are taken into account quantitatively, SID_a _- SID_e _should equal 0 (electrical charge neutrality) unless there are unmeasured charges to explain this 'ion gap'. Such charges are described by the strong ion gap (SIG):

SIG = SID_a_- SID_e_

A positive value for SIG must represent unmeasured anions (such as sulfate, oxo acids, citrate, pyruvate, acetate and gluconate) that must be included to account for measured pH. The traditional anion gap was also calculated as anion gap = [Na^+^] + [K^+^] - [Cl^-^] - [HCO_3_^-^], with a reference range of 12–20 mmol/l [20].

Data are expressed as means ± s.d., or as percentage. Student's *t*-test was used to compare the study group and the comparison group (StatView; Abacus Concepts, Berkeley, CA, USA). *P *< 0.05 was considered statistically significant.

## Results

One hundred and five patients with out-of-hospital cardiac arrest were included in this study. The demographics of these patients are presented in Table 1. They had a mean age of 62.2 years and included 75 (71%) males and 30 (29%) females. Most of the patients had an initial rhythm of asystole (54%) or pulseless electrical activity (38%), and the number of witnessed arrests was 10 (10%). The main cause of collapse was cardiogenic (57%), followed by trauma (12%) and hanging (9%); 19% of the patients had a return of spontaneous circulation.

These 105 patients were compared with 28 patients with minor injuries as a comparison group (mean age 40.2 years; 19 males and 8 females). The mean interval from arrival at the emergency room to blood sampling was similar between the two groups (cardiac arrest, 12.9 ± 10.3 min; minor injury, 12.3 ± 5.5 min; *P *= 0.78).

The acid–base variables in cardiac arrest and minor injuries are shown in Table 2. Except for sodium and SID_a_, all variables were significantly different between the two groups. In brief, patients with cardiac arrest were acidemic (pH 6.90 versus 7.39; *P *< 0.0001), secondary to metabolic acidosis (standard base excess -19.1 versus -1.5 meq/l; *P *< 0.0001) compared with the comparison group. Patients with cardiac arrest were also hyperkalemic, hypochloremic, hyperlactatemic and hyperphosphatemic. The anion gap and SIG were also higher in patients with cardiac arrest.

**Figure 1:**
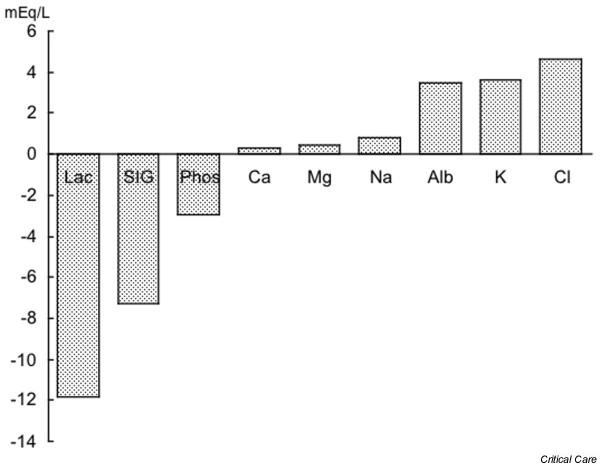
The impact of each variable on the acid–base status of patients with out-of-hospital cardiac arrest. Each value is presented as the difference between the mean for the comparison group and the study group. A negative value suggests an acidifying effect, and a positive value an alkalinizing effect. Alb, albumin; Ca, calcium; Cl, chloride; K, potassium; Lac, lactate; Mg, magnesium; Na, sodium; Phos, phosphate; SIG, strong ion gap.

Figure 1 shows the acid–base impact of each variable in patients with cardiac arrest compared with the comparison group. Lactate was the strongest determinant of acidemia, accounting for -11.8 meq/l of acidifying effect. However, SIG contributed -7.3 meq/l of acidifying effect and phosphate -2.9 meq/l. This acidemia was attenuated by the alkalinizing effect of several variables. A decrease in chloride had the strongest alkalinizing effect (+4.6 meq/l), followed by an increase in potassium (+3.6 meq/l) and a decrease in albumin (+3.5 meq/l).

## Discussion

It has been known for decades that patients with cardiac arrest invariably develop a severe metabolic acidosis [1-6,21-23]. This acidosis has been thought secondary to hyperlactatemia [2]. However, the correlation between standard base excess and lactate has been reported to be poor, suggesting that other factors might participate in the pathogenesis of cardiac arrest acidosis [3]. The problem is that no previous studies quantitatively analyzed the cause of metabolic acidosis in these patients. We therefore sought to define and quantify acid–base status in these patients by applying the quantitative principles of acid–base analysis described by Stewart, Figge and colleagues [13,14]. Using this methodology, we found that the causes of acidosis were much more complex than previously thought. Although lactate was the biggest contributor to metabolic acidosis and the development of acidemia in these patients, it accounted for only about 50% of it, whereas SIG and phosphate combined contributed an almost equal percentage (about 33% and 13%, respectively). However, this acidosis was associated with strong compensating responses, which attenuated its severity. These responses included hypochloremia, hyperkalemia, hypoalbuminemia and, to a smaller extent, hypermagnesemia and hypercalcemia.

A key finding was that patients with cardiac arrest had a disproportionately higher SIG than the comparison group (12.4 versus 5.1 meq/l; *P *< 0.0001). Although the source of this disproportionate increase in unmeasured anions was not specifically addressed, possible candidates include sulphate, urate, oxo acids, amino acids and other organic acids. Kaplan and colleagues reported increased SIG in patients with major vascular injury, another type of global tissue hypoperfusion [24]. It therefore seems that unmeasured anions are likely to be generated during global tissue hypoxic states.

Hyperphosphatemia in patients with cardiac arrest has been underemphasized as a contributor of acidosis (2.95 mmol/l in our study patients). Although causes of this abnormality remain unclear, transcellular shift, cellular injury and phosphate release might be responsible [25,26]. Hyperphosphatemia is also related to other types of metabolic acidosis [27,28]. However, because phosphate is not included in calculations of the anion gap, its impact on acid–base status is often poorly appreciated. The Stewart–Figge methodology can reveal its importance. For example, we previously reported that, in patients with acute renal failure, hyperphosphatemia accounted for about 20% of the difference in the acid–base status of patients compared with controls [29].

These acidifying effects were partly attenuated by a concomitant metabolic alkalosis, due mainly to hypochloremia, hyperkalemia and hypoalbuminemia. Their alkalinizing effects in cardiac patients were 4.6, 3.6 and 3.5 meq/l, respectively. The alkalinizing effects of hypercalcemia and hypermagnesemia accounted for less than 1 meq/l of alkalinizing effect. These abnormalities in four serum electrolytes and albumin can be explained by transcellular electrolyte shifts and, perhaps, previous co-morbidities. The existence of metabolic alkalosis in patients with cardiac arrest is not intuitive, unless each element is quantified and compared with a control.

There are several limitations in this study. First, only 10% of patients had their arrest witnessed and most of patients had an initial rhythm of asystole or pulseless electrical activity. Furthermore, drug injection by the emergency ambulance service is not approved in Japan, which inevitably delays the start of advanced life support. Our results might therefore not be applicable to patients in other institutions or countries. However, published clinical studies examining acidemia during cardiac arrest in a quantitative manner are lacking, particularly in terms of patients who have no received advanced life support interventions. Our study presents the first quantitative acid–base analysis for this disorder. Furthermore, the lack of drug or fluid administration in the field provides a unique opportunity to study these disorders with minimal iatrogenic modifications.

Second, we used patients with minor injuries as a comparison group. Although they were well enough to be discharged from the hospital within 2 days, mild hyperlactatemia (2.5 mmol/l) was present in these patients. However, all other variables, including pH and bicarbonate, were in the normal ranges. Considering the large difference in lactate between the two groups (11.8 mol/l), this group of patients, although imperfect, seems adequate for comparison.

Third, fluid resuscitation had started just before or at the time of blood sampling in some patients. This might have affected acid–base status in both groups. Unfortunately, we did not collect precise information on such fluid administration (timing, amount or number of patients so treated) because of the logistic difficulty of collecting such detailed information while attempts were being made to save the life of the patients. This is a significant limitation of our study because some of the fluid given might have affected our findings. However, blood sampling was conducted 12 min on average after admission to the emergency room in both groups, and only small amounts of fluid resuscitation (acetate Ringer in both groups) would have been given to these patients before sampling. Although SIG might have been somewhat affected by acetate in the fluids, the difference in the amount of acetate Ringer given before blood sampling is unlikely to have been large enough to fully explain the significant difference in SIG between the two groups.

The last limitation was that, although we attempted to collect arterial samples, it was often difficult to distinguish which sample, arterial or venous, was actually collected from patients with cardiac arrest. Some of the differences we found might therefore have been due to the higher incidence of venous sampling in the cardiac arrest group. These difficulties are inherent in research in the emergency setting.

## Conclusion

Using the Stewart–Figge methodology, we studied the acid–base status of out-of-hospital cardiac arrest patients and found its pathogenesis to be complex. We found that lactate accounts for only 50% of the metabolic acidosis and consequent acidemia seen in such patients and that an increase in unmeasured anions and phosphate accounts for the rest. We also found that their acidifying effect was partly attenuated by the alkalinizing effect of hypochloremia, hyperkalemia, hypoalbuminemia, hypermagnesemia and hypercalcemia. The clinical and prognostic significance of these changes requires further investigation.

## Key messages

• 	Lactate accounts for only 50% of metabolic acidosis in cardiac arrest, and SIG and phosphate combined contributed almost an equal percentage.

• 	This acidosis is attenuated by hypochloremia, hyperkalemia and hypoalbuminemia.

• 	acid–base status in patients with cardiac arrest is more complex than previously thought.

## Abbreviations

*P*_CO2_, partial pressure of CO_2_; SID_a _= apparent strong ion difference; SID_e _= effective strong ion difference; SIG = strong ion gap.

## Competing interests

The author(s) declare that they have no competing interests.

## Authors' contributions

JM and SU conceived the study, designed the trial and supervised the conduct of the trial and data collection. HM provided statistical advice on analyzed data, and RB chaired the data oversight committee. JM drafted the manuscript, and all authors contributed substantially to its revision. JM takes responsibility for the paper as a whole. All authors read and approved the final manuscript.

## Figures and Tables

**Table 1:** Demographics of patients with cardiac arrest

Parameter	Value
Age (years)	62.2 ± 15.5
Male (%)	75 (71%)
Arrest witnessed (%)	10 (10%)
Initial rhythm (%)	
Asystole	57 (54%)
Pulseless electrical activity	40 (38%)
Ventricular fibrillation	8 (8%)
Cause of arrest (%)	
Cardiogenic	60 (57%)
Trauma	13 (12%)
Hanging	9 (9%)
Respiratory	4 (4%)
Neurological	4 (4%)
Other	13 (12%)
ROSC (%)	20 (19%)

ROSC, return of spontaneous circulation.

**Table 2:** acid–base variables in patients with cardiac arrest and with minor injuries

Variable	Cardiac arrest	Minor injury	*P*
pH	6.90 ± 0.21	7.39 ± 0.08	0.0001
*P*_CO2 _(Torr)	78.3 ± 42.8	39.2 ± 9.1	0.0001
Bicarbonate (mmol/l)	13.8 ± 5.4	22.8 ± 3.5	0.0001
Standard base excess (mmol/l)	-19.1 ± 6.2	-1.5 ± 3.6	0.0001
Sodium (mmol/l)	140.4 ± 5.9	139.6 ± 2.8	0.49
Potassium (mmol/l)	7.3 ± 2.6	3.6 ± 0.4	0.0001
Ionized calcium (mmol/l)	1.31 ± 0.12	1.17 ± 0.08	0.0001
Total magnesium (mmol/l)	1.17 ± 0.27	0.86 ± 0.14	0.0001
Chloride (mmol/l)	98.6 ± 5.9	103.0 ± 4.6	0.0002
Lactate (mmol/l)	14.3 ± 5.8	2.5 ± 1.8	0.0001
Albumin (g/dl)	3.4 ± 0.7	3.9 ± 0.5	0.0002
Phosphate (mmol/l)	2.95 ± 1.07	1.06 ± 0.36	0.0001
Anion gap (meq/l)	20.1 ± 7.4	11.0 ± 3.5	0.0001
SID_a _(meq/l)	38.9 ± 4.6	41.0 ± 2.9	0.22
SID_e _(meq/l)	26.5 ± 6.1	35.9 ± 4.0	0.0001
Strong ion gap (meq/l)	12.4 ± 6.0	5.1 ± 3.6	0.0001

*P*_CO2_, partial pressure of CO_2_; SID_a_, apparent strong ion difference; SID_e_, effective strong ion difference.
